# News and Miscellany

**Published:** 1901-03

**Authors:** 


### News and Miscellany.

**At this date** (March 6th) the bill for a State Board of Health, adopted by the State Medical Association, has not been introduced in either house. The committee having it in charge have not put in an appearance so far.

**An Act** for compulsory vaccination was introduced in the house this wee.k It is meeting with fanatical opposition.

---

**Dr. J. M. Hons,** of San Marcos, is at New Orleans Polyclinic and wants the Red Back sent to him there; can't afford to miss a copy.

---

**The last Straw.**—I have received a letter addressed to "Dr. F. E. Douill." Such is fame; die for your country—and have your name spelled wrong in the papers.

---

**Raised Them.**—The Senate has passed a bill raising the salary of asylum superintendents from $2000 to $3000; and of assistant physicians from $1200 to $1800; all "find" themselves.

---

**A Harmony of Sweet Sounds** may be expected at the next meeting of the American Congress of Tuberculosis. Dr. A. N. Bell is President; Dr. Raley H. Bell is Vice-President, and Hon. Clark Bell is Secretary.

---

**Change of Address.**—Dr. D. M. Blair, from Rosenberg to Lufkin, Texas. Dr. J. A. T. Paige, from Nesbit to Wortham, Texas. Dr. E. J. Burns, from Giddings to Oscar, Texas. Dr. H. L. Wilder, from Francis, O. T., to Dallas, Texas. Dr. Bat. Smith, from Wharton to Matthews, Texas.

---

**Medico=Legal Surgery.**— Section, Medico-Legal Surgery and Section in Railroad Surgery, will hold a joint session with the Medico-Legal Society in New York, third Wednesday in April, 1901. A big dinner will be given on the occasion. Railroad Surgeons are invited to become members of the association; to contribute papers, and to help eat the dinner. Address Dr. Chas. K. Cole, Helena, Mont., Chairman Section, or Clark Bell, Esq., Sec., 39 Broadway, N. Y., for further information.

---

**Dr. Willis P. King,** whose paper on the lymph treatment appears in this issue, is well and favorably known to the profession at large. He has been First Vice-President American Medical Association: President Missouri State Medical Association; Chief Surgeon M., K. & T. R. R., and Professor of Medicine in one of the Kansas City Medical Colleges. He is also well known as the author of that charming little work: Stories of a Country Doctor. His paper should receive careful attention and be widely read.

**New Orleans Polyclinic.**—Physicians will find the Polyclinic an excellent means for posting themselves upon modern progress in all branches of medicine and surgery. The special ties are fully taught, particulary laboratory work. Fourteenth annual session opens November 12, 1900. For further information address Dr. Isadore Dyer, Secretary, New Orleans Polyclinic, New Orleans.

---

**On January 12th** the resignations of Drs. Milliken, Titterington and Ashton were accepted by the Faculty of the Medical Department, University of Dallas, and the vacancies filled as follows:

Professors of Surgery, Drs. B. E. Hadra, J. M. Inge and Joe Becton.

Professor of Opthalmology, Dr. Theo. Arnold.

Professor of Practice of Medicine and Dean of the Faculty, Dr. C. M. Rosser.

---

**Getting in their Work; Making Hay** while the lamp holds out to burn—those sinners are. That is to say, several members of one of the District Medical Examining Boards from North Texas are in New Orleans, licensing the undergraduates of the Tulane at $15 a license, hurrying up things before the new medical practice act can go into effect. It just can't be helped, it seems, and there is no way of undoing their nefarious work. Poor Texas—long suffering Texas, when will she enter the New Jerusalem so long seen by faith; so near, yet so far.

---

**The Quarantine Law Amendment.**—Dr. Blunt, the State Quarantine Officer, is seeking to have the quarantine law amended so as to remedy the defects to which this JOURNAL called attention last fall, which defects rendered it questionable if counties could be made to institute quarantine and pay expenses of epidemics. The status of the bill which is, at this writing, before the house, is—it has been so amended as to make the county pay one-third of the expense of a local epidemic and the State, two-thirds. It has passed to engrossment and will be put upon its passage, in the house, at an early date.

---

**Dr. Raley Husted Bell,** so well and favorably known to the medical profession through his medical journal work on "The Magazine of Medicine," formerly published by him at Atlanta;

"The Raven," more recently issued by him (St. Louis), and his charming little volume of poems entitled *Aala Deane, and other Poems*, is now on the staff of the *Medico Legal Journal* as associate editor, with Judge Clark Bell the distinguished editor and founder of the valuable publication, and founder and long time President of the Medico-Legal Society. The *Medico Legal Journal*, and its hosts of readers, are to be congratulated upon the acquisition of so vigorous and versatile a writer as Dr. Raley H., and we may look to see at once the effects of this infusion of new blood into its already strong veins.

